# Use of the FebriDx point-of-care test for lower respiratory tract infections in primary care: a qualitative interview study

**DOI:** 10.3399/BJGPO.2024.0024

**Published:** 2024-08-21

**Authors:** Jill Rutter, Christopher R Wilcox, Nour Odeh, Ingrid Muller, Tristan W Clark, Paul Little, Firoza Davies, John McGavin, Nick Francis

**Affiliations:** 1 Primary Care Research Centre, University of Southampton, Southampton, UK; 2 School of Clinical and Experimental Sciences, University of Southampton, Southampton, UK; 3 National Institute for Health and Care Research Southampton Biomedical Research Centre, Southampton, UK; 4 Primary Care Research Centre, University of Southampton, Southampton, UK

**Keywords:** infectious illness, qualitative research, diagnosis, point-of-care testing

## Abstract

**Background:**

FebriDx is a single-use, analyser-free, point-of-care test with markers for bacterial (C-reactive protein [CRP]) and viral (myxovirus resistance protein A [MxA]) infection, measured on a finger-prick blood sample.

**Aim:**

As part of a larger feasibility study, we explored the views of healthcare professionals (HCPs) and patients on the use of FebriDx to safely reduce antibiotic prescriptions for lower respiratory tract infections (LRTIs) in primary care.

**Design & setting:**

Remote semi-structured qualitative interviews were conducted in South England.

**Method:**

In total, 22 individuals (12 patients who underwent FebriDx testing and 10 HCPs from general practices that conducted testing) participated in interviews, which were analysed thematically.

**Results:**

Patients and HCPs expressed positive views about use of the test. They felt FebriDx was a useful tool to inform prescribing decisions and provided a visual aid to support shared decision making and appropriate antibiotic use. Most felt it would be feasible to integrate use into routine primary care consultations. Some practical difficulties with blood collection and interpreting results, which impacted on usability, were identified. Some patients’ reactions to negative test results suggested the need for better communication alongside use of the test.

**Conclusion:**

FebriDx was perceived as a useful tool to guide antibiotic prescribing and support shared decision making. Initial practical problems with testing and communicating results are potential barriers to use. Training and practice on using the test and effective communication are likely to be important elements in ensuring patient understanding and satisfaction, and successful adoption.

## How this fits in

FebriDx is a CE-marked, single-use, point-of-care test with markers for bacterial and viral infection. To the authors’ knowledge, no previous studies have explored the views of patients or clinicians on its use. FebriDx was perceived as a useful tool to guide antibiotic prescribing and support shared decision making. Practical problems with testing and communicating results are potential barriers that need to be addressed in training to ensure successful adoption and patient satisfaction.

## Introduction

There is a drive to safely reduce antibiotic use in primary care.^1–3^ Acute lower respiratory tract infections (LRTIs) are commonly managed with antibiotics, despite most being of viral aetiology and/or self-limiting.^1,4,5^ One of the challenges is that bacterial and viral LRTIs can be difficult to differentiate clinically. Rapid diagnostic testing at the point of patient contact (‘point-of-care testing’ [POCT]) may help to reduce unnecessary antibiotic use.^6–9^ POCT has been broadly well received in recent studies, but is yet to be widely adopted into routine UK primary care.^10–13^ Such devices typically detect a single host response biomarker or pathogen (typically C-reactive protein [CRP], influenza, or severe acute respiratory syndrome coronavirus 2 [SARS-CoV-2]), which may be challenging to interpret and/or act on in isolation.^6,8,9^ Furthermore, many devices require a ‘desktop’ analyser, which are often impractical for the primary care setting and incur high up-front costs and maintenance requirements.^6,8,9,14,15^

FebriDx^16^ is a CE-marked, single-use, analyser-free POCT device. It detects two host response proteins, CRP and myxovirus resistance protein A (MxA), using finger-prick blood, with results available after 10 minutes. CRP is an acute phase reactant that generally increases to higher levels with bacterial compared with viral infection, and MxA is stimulated by interferon α and β, and associated with viral infection.^17–19^ As a portable, dual-marker test, FebriDx may therefore be more clinically helpful and practical to use compared with other POCT devices in the primary care setting.

Several recent studies in the UK and US have explored its use as a triage tool for COVID-19 in emergency departments; however, there is very limited data on its impact on antibiotic prescribing^20–28^ and only one small retrospective study from a single UK GP practice has examined the use of FebriDx in primary care.^14^ Furthermore, to the authors’ knowledge, no studies have explored the views of patients or clinicians on FebriDx use, yet a lack of feedback and input from clinicians into the design of current POCT devices has been frequently cited as a barrier to uptake.^6,8,9^

The aim of this qualitative interview study (nested within a larger feasibility study) was therefore to explore the views of primary care HCPs and patients on the use of FebriDx to safely reduce antibiotic prescriptions for LRTIs in primary care.

## Method

### Study design

This was a qualitative interview study with HCPs and patients in general practice. An exploratory literature review was conducted in December 2021 to inform the development of the study protocol and interview topic guides (see Supplementary Information S1). Remote semi-structured interviews were undertaken from February–July 2023.

### Context

This qualitative interview study was nested within the PREFIX study (Point of care testing using FebriDx to improve antibiotic use for respiratory tract infections in primary care).^29^ The PREFIX study was a prospective, multi-centre, feasibility study undertaken from January–June 2023, and explored the usability and potential impact of FebriDx in reducing antibiotic prescriptions for LRTIs in primary care. Patients presenting with an LRTI were invited to participate following initial clinical assessment if a prescribing clinician deemed that they would be likely to prescribe antibiotics in the absence of further diagnostic testing. Participants were recruited at nine general practices in South England, and 162 participants underwent FebriDx testing.

**Figure 1.**
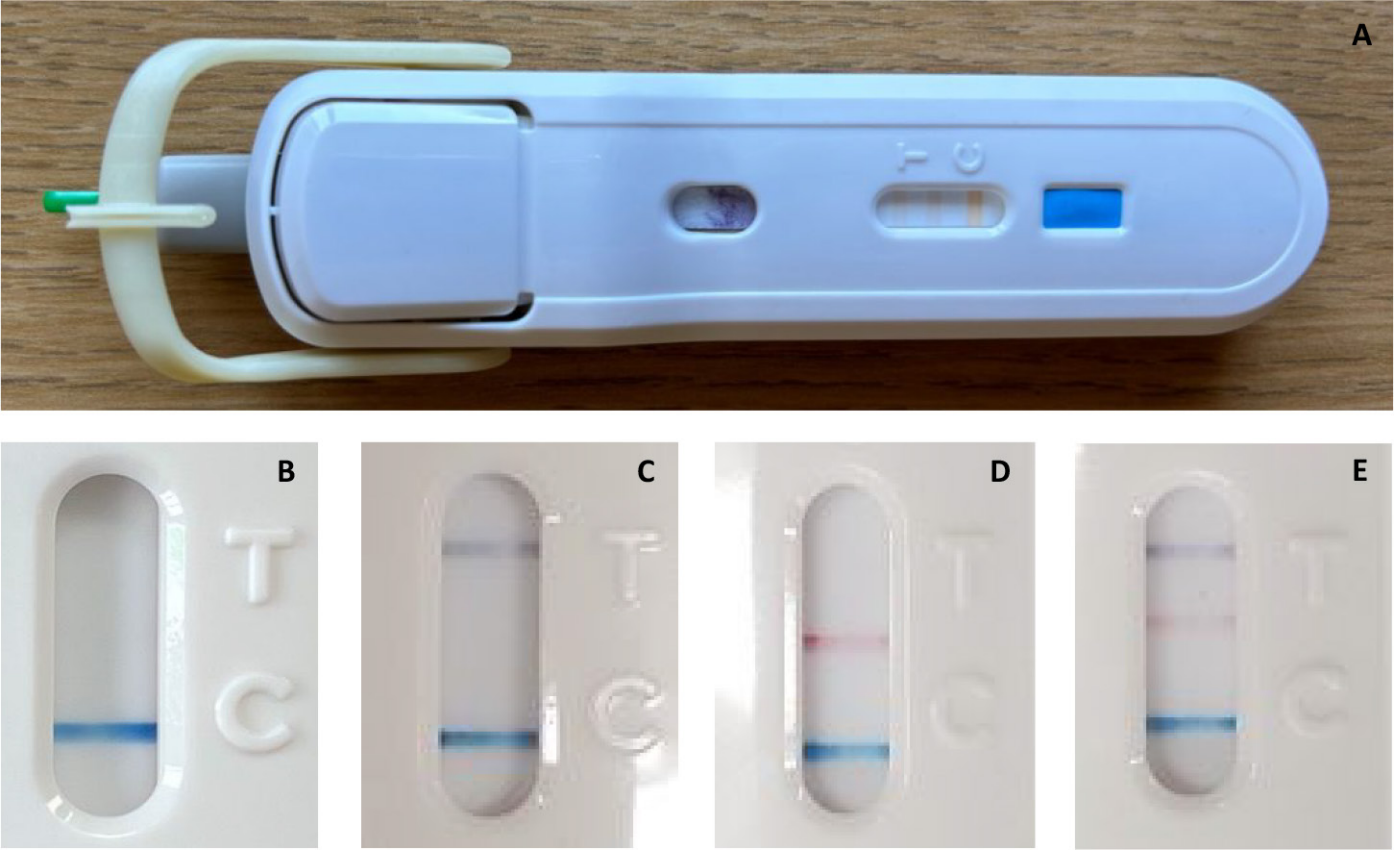
The FebriDx device and its possible results. **A**) The FebriDx device; **B**) a negative result with a blue control line; **C**) a grey line indicating positive C-reactive protein (CRP) (lower limit of detection [LLoD] 20 mg/l); **D**) a red line indicating positive myxovirus resistance protein A (MxA) (LLoD 40 ng/ml); and **E**) all lines present indicating both CRP and MxA positive. The presence of a grey CRP line with no red MxA line is suggestive of a bacterial infection. A red MxA line is suggestive of a viral infection. The presence of both CRP and MxA is suggestive of viral infection, but doesn’t exclude a concurrent bacterial infection. A negative test result (no grey or red lines) indicates an undetectable level of either CRP or MxA. In most cases, this suggests a lack of significant host response to infection, most likely because the illness is minor or the patient is in the recovery phase of their illness.

The FebriDx test involves a finger-prick blood test, and results are displayed as three lines on a lateral flow strip (Figure 1). Verbal and written guidance on the use and interpretation of FebriDx was provided to all sites, and clinicians were advised that any decision should take into account both FebriDx results and clinical judgement.

### Recruitment

A purposive sampling approach was undertaken to select a range of participants based on age, sex, ethnicity, site, test result, and clinical role (if applicable). Patients were eligible if they had taken part in the PREFIX study and consented to be invited to a follow-up interview. In the case of children, parents consented to be invited. HCPs were eligible if they had a role in administering and/or interpreting FebriDx testing, and/or communicating the results to patients as part of the PREFIX study. HCPs were invited via email, as well as by snowball sampling. All participants were offered a 40 GBP voucher as compensation.

### Data collection

After taking informed consent from participants (recorded verbal consent), semi-structured interviews^30^ were conducted (remotely) by one qualitative researcher (JR) using topic guides, which were refined after initial piloting (see Supplementary Information S1). The topic guides included questions about experiences of FebriDx testing in this study, and its potential use in routine care. Topic guides were developed in collaboration with the wider study team, including public contributors. Patient interviews were conducted from February–June 2023, and HCP interviews from April–July 2023. All interviews were audio-recorded, transcribed, and anonymised.

### Data analysis

Thematic analysis was undertaken using an inductive, constant–comparative approach.^31,32^ JR initially gained familiarisation with the transcripts and checked them against the audio-recordings. Transcripts were coded using NVivo (version 1.6.1). When new codes emerged, earlier transcripts were re-analysed to look for these codes. Interviews were coded by a single researcher (JR) and organised into sub-themes. Interview transcripts were additionally reviewed by the wider study team (CW, NO, IM, and NF), and initial codes and sub-themes were refined after discussion as a group. After 22 interviews, recruitment ended as it was felt that there was replication of codes and sub-themes, with minimal emergence of new codes and sub-themes.^33^ Data were reported according to the Standards for Reporting Qualitative Research checklist.^34^

JR gained familiarisation with transcripts and coded the narrative into units of meaning. Emerging codes were scrutinised for patterns, similarities, differences, contradictions, and observations, which led to groups of codes and themes being generated. A coding framework was developed by placing each item of coded data in a named category in the framework. Initial codes and themes were discussed with the study team and refined.

### Patient and public involvement

Two White British men aged 72 and 75 years, and an Asian British female aged 44 years (mother of a young child), attended study meetings and had input into the study design, topic guides, data interpretation, and dissemination.

## Results

### Participants

In total, 22 participants were interviewed from six of the nine general practices involved in the PREFIX study: 12/162 (7%) patients (nine adult patients and three parents of child patients) and 10/31 (32%) HCPs (Table 1). A summary of recruiting general practice sites is displayed in Supplementary Table S1. The mean age of adult patients was 67 years (range 50–84 years), and the mean age of children was 5 years (range 3–7 years). The mean age of HCPs was 46 years, and mean time since qualification was 14 years (data not available for one HCP). Five of the HCPs were research nurses, one was a research administrator, and four were GPs. Mean interview length was 42 minutes (range 26–54 minutes).

**Table 1.** Study participants

Study ID	Practice ID	Age, years	Sex	Ethnicity	Occupation (years qualified)	Prior research participation	Test result	Antibiotic prescription
**Patients (** ***n*** **= 12)**								
01-001-004	1	68	M	White British	Logistics manager	Yes	CRP positive MxA negative	Yes
02-001-005	1	64	M	White British	IT consultant	Yes	CRP negative MxA negative	No
03-002-003	2	63	F	White British	Retired nurse	Yes	CRP positive MxA negative	Yes
04-001-003	1	74	M	White British	Teacher	Yes	CRP positive MxA negative	Yes
05-001-011	1	65	M	White British	Accountant	Yes	CRP negative MxA negative	Yes (delayed)
06-001-009	1	5 (mother 31)	M	White British	Domiciliary care worker	No	CRP positive MxA negative	Yes
07-001-008	1	77	F	White British	Healthcare support worker	Yes	CRP negative MxA negative	No (Yes after reconsultation)
08-005-005	5	84	F	White British	Pharmacy manager	Yes	CRP positive MxA negative	Yes
09-005-006	5	61	F	White British	Intermediate care worker	Yes	CRP positive MxA negative	No (Yes after reconsultation)
10-003-012	3	50	F	White British	Teaching assistant	Yes	CRP negative MxA negative	No
11-003-013	3	7 (mother 47)	M	White British	Therapist	No	CRP negative MxA negative	No
12-003-022	3	3 (mother 39)	F	Black	Child social worker	No	CRP negative MxA negative	No
**Practice staff (** ***n*** **= 10)**								
RN-01–001	1	35	F	White British	Research nurse (unknown)	No	N/A	N/A
GP-02–001	1	61	F	White British	GP (21)	Yes	N/A	N/A
RN-03–004	4	52	F	White British	Research nurse (19)	No	N/A	N/A
GP-04–003	3	45	F	White British	GP (4)	No	N/A	N/A
RN-05–002	2	44	F	White British	Prescribing research nurse (22)	Yes	N/A	N/A
RA-06–006	6	56	F	White British	Research administrator (1)	No	N/A	N/A
GP-07–004	4	56	F	White British	GP (16)	Yes	N/A	N/A
RN-08–003	3	49	F	White British	Research nurse (25)	No	N/A	N/A
RN-09–006	6	32	F	White British	Research nurse (11)	No	N/A	N/A
GP-10–006	6	34	M	White British	GP (4)	Yes	N/A	N/A

CRP = C-reactive protein. F = female. M = male. MxA = myxovirus resistance protein A. N/A = not applicable.

Nine sub-themes were developed from the data and grouped under three themes (Table 2).

**Table 2.** Themes and sub-themes identified from the thematic analysis

Theme	Practicalities of FebriDx use
Sub-theme 1	Obtaining blood samples
Sub-theme 2	Suitability for different age groups
Sub-theme 3	Reading FebriDx results
Sub-theme 4	Integrating FebriDx into consultations
**Theme**	**Interpreting and communicating results**
Sub-theme 5	Confidence in FebriDx results
Sub-theme 6	Reactions to FebriDx results
Sub-theme 7	Communication of FebriDx results
**Theme**	**Impact of FebriDx on decision making**
Sub-theme 8	Facilitating shared decision making
Sub-theme 9	Supporting appropriate antibiotic use

### Theme 1: Practicalities of FebriDx **use**

#### Obtaining blood samples

Patients were generally not concerned about having the test done (including the finger prick), especially if they were used to having tests for long-term conditions (such as diabetes). They valued knowing their blood sample would inform a treatment decision:

*'I've got no great problems with any needles of any sort and I've had some fairly big ones*. […] I *don't even remember it happening, it was so inconsequential.'* ([Patient] PT-05-001-011)

Some patients and HCPs reported practical difficulties in obtaining an adequate blood sample, often owing to not getting enough blood, or issues with transferring blood onto the test strip. In some cases, devices were discarded and a new device used.

*'The bit that I've struggled most with, actually getting the blood* […] *I can often get quite a big drop of blood out of the patient, but it’s trying to get it into the tube. It looks like it’s gone virtually all the way down the tube, and then you flick it over, and it doesn't then dispense on to the test strip.'* (GP-04-003)

*'It needs to be like triple the size*. […] *that collection point either needs to be scrapped or changed … '* (PT–11-003-013, parent of 7 year old)

#### Suitability for different age groups

Most patients and HCPs thought FebriDx was suitable for use in a range of patients, especially for older people where there was felt to be a particular tendency to overprescribe antibiotics. Some patients and HCPs raised concerns about FebriDx use in very young children:

*'In a child her age, the pricking part was really difficult for her, and she was upset for some few minutes* […] *then okay, after.'* (PT–12-003-022 [parent of 3-year old])

#### Reading FebriDx results

Some HCPs found it difficult to interpret test results when the lines were perceived as being faint. Others reported finding it straightforward, particularly as they had similar experiences interpreting COVID-19 lateral flow tests:

*'Some of them were* […] *really, really faint. It took two of us peering at it.'* (GP-02-001)

*'Reading the results was dead easy because it’s the same as all these other COVID tests and* […] *it’s the same as all the other tests where you wait for a control line, and then you know whether it’s positive or not.'* (GP-07-004)

#### Integrating FebriDx into consultations

Most patients found having a FebriDx test convenient and thought it was an easy test to undergo. They commented that compared with using laboratory tests it was quick to get the results and decide on appropriate treatment. Furthermore, many were comfortable with the idea owing to familiarity with COVID-19 lateral flow tests, and most patients expressed no concerns about having to wait for results.

HCPs liked the idea of the FebriDx test in general and most felt that testing was straightforward and could be introduced into patient pathways:

*'I think there are lots of good reasons to bring it into practice. I don’t think it’s a burden, it doesn’t take that long. It could reduce people coming back because they’ve had the right treatment. I think there are more positives than negatives.'* ([Nurse] RN-09–006)

*'I think obviously if it weren’t part of a trial* […] *it would be very convenient because you could just plan that ten-minute gap and* […] *then be ready to see the patient.'* (RN-01–001)

The logistics of FebriDx use (and who performed the test) varied between practices. Some HCPs raised concerns about the impact on consultation time in a traditional clinic setting where patients have set appointment times, but thought it was more feasible to integrate the POCT into a ‘*duty doctor*’ or ‘*sit-and-wait*’ service:

*'It takes ten minutes to get the results […] so it doesn't fit into a ten-minute consultation!'* (GP-04-003)

*'My overall feeling is it’s a brilliant idea, brilliant concept, but to make it work in general practice* […] *is to make it easier, less blood, and make it quicker* [… It would be] *good in a duty doctor situation* [… when] *you're given a time but it’s just an indication of what time you may be seen.'* (RN-08–003)

Some HCPs described how FebriDx testing (and communication of the results and management plan) worked efficiently when delegated to nursing staff after an initial GP consultation:

*'*[The patient will] *wait in the waiting room while the test is being performed, and once I've got the results, I call the clinician who initially spoke to them* [… and then I’ll] *bring the patient back* […] *and tell them what the doctor said.'* (RN-03–004)

One HCP suggested doing the test at the start of the consultation, so the results would be ready by the end of the consultation to help them make a final decision:

*'I almost feel like it’s useful to have the result at the end of your normal consult*. […] *when the patient comes in* […] *we'd like to do this test, to help support our decision making, and confirm whether we feel there’s a virus or bacterial infection* [… then you do] *your usual consult,* [… and at the end] *you'll have a result through to review with the patient*.*'* (GP-10–006)

### Theme 2: Interpreting and communicating results

#### Confidence in FebriDx results

Most patients seemed to have a high level of confidence in FebriDx and took the results at face value without questioning their accuracy, often owing to experience with COVID-19 testing:

*'Patients were very believing of them. I thought there would be scepticism, or, "How do you know it’s true?" Actually, I think they’ve been pre-empted by the fact they’ve had the COVID test.'* (GP-02–001)

Conversely, there appeared to be some variation among clinicians about whether the test should be used as a tool to aid decision making, or be viewed as a definitive answer:

*'The clinicians approach it very differently. Some* […] *whatever the test says, they're very likely to do that* […] *others will be more, "Well, the test says this, but I still think this."'* (RN-01–001)

#### Reactions to FebriDx results

Patients who had a test result that was suggestive of bacterial infection (CRP positive, MxA negative) often felt pleased as it validated how they were feeling and meant that ‘something could be done for them’:

*'The fact it was a bacterial infection, in a strange way it was quite pleasing really.'* (PT-04-001-003)

On the other hand, reactions to negative test results (that is, both CRP and MxA negative) were more variable. Some felt reassured as it meant they wouldn’t receive unnecessary antibiotics, while others had quite strong emotional reactions of disappointment, confusion, and/or scepticism. Often, these negative views were associated with a belief that there was nothing medically that could be done for them, or a feeling of powerlessness:

*'I really couldn't believe it*. […] *You know your own body, and you know when you really feel that you need some sort of help, don’t you?'* (PT-07-001-008)

*'I thought that I've had it*. […] *What do they do for people if you can't have antibiotics?'* (PT-09-005-006)

*'Maybe I'd been hoping that it was an infection that I could have then remedied with some penicillin.'* (PT-05-001-011)

#### Communication of FebriDx results

There seemed to be a degree of mismatch between the perceptions of HCPs and patients regarding communication of results, particularly negative results. HCPs emphasised the importance of listening to patients' concerns, being very clear with patients about the purpose of the FebriDx test, and the implications of the results before undergoing testing:

*'You just have to listen to your patient really and explain everything really thoroughly and carefully and tell them what you're doing* […] *I will always tell them before we do the test that it will either be bacterial or viral or nothing, so I do pre-warn them that there may not be a positive test result, but that doesn’t mean that they’re not poorly and there’s nothing wrong with them.'* (RN-03–004)

*'I felt it was really important that they understood that I could change my mind clinically. I think getting them to really understand that was really important. You had to have buy-in with them.'* (RN-05–002)

On the other hand, some patients felt communication of negative test results was poor, that their concerns had not been taken seriously, and that the test was used to dismiss their worries.

Patient: *'The results came back negative, so no bacterial infection and no viral infection*.*'*

Interviewer: *'How did she explain the results?'*

Patient: *‘Well, she couldn't* […] *I think the biggest issue as I see it is that instead of using it as a tool* […] *to see what is wrong with a patient, it could be used as a tool to dismiss a patient* […] *especially if it comes back negative*.*'* (PT-02-001-005)

*'*[The HCP said] *"Well, it’s negative," and I said, "You're joking"* […] *I just felt so unwell. Then they just said, "Well* […] *just carry on doing what you've been doing."'* (PT-07-001-008)

### Theme 3: Impact of FebriDx on decision making

#### Facilitating shared decision making

Particularly owing to experience with COVID-19 tests, both patients and HCPs perceived FebriDx as a useful visual aid to facilitate shared decision making:

*'I would tend to show them the kit itself* […] *I think patients quite enjoyed being involved* […] *It also gives them the visual cue* [… and] *the decision making is being supported* […] *It’s quite a nice way to involve the patient.'* (GP-10–006)

Especially in cases where patients were expecting an antibiotic (or seemed sceptical about relying on clinical signs alone), HCPs described that being able to show the line on the FebriDx device improved patient’s trust in their decision making, as well as their own confidence:

*'If you can show that it’s not actually a bacterial infection, I think it’s quite useful* […] *I think* […] *that some of them may have pushed harder to have antibiotics if it wasn’t for having the test* […] *so I think it’s worked for our confidence and the patients’ confidence.'* (GP-04–003)

*'She said, "Look, you can see it’s one line. It’s a viral rash"* […] *So we knew he didn't need antibiotics* [… it was great to have] *more backup* [rather than] *just from someone’s opinion.'* (PT11-003-013 [parent of 7-year old])

*'There are definitely patients that it’s useful in* [... especially] *if patients are not confident in what you're saying about the viral infections* [...] *so sometimes it’s for our confidence, but sometimes it’s for the patient’s confidence.'* (GP-04–003)

#### Supporting appropriate antibiotic use

Most patients and HCPs had positive views about use of FebriDx with regard to helping reduce the overuse of antibiotics:

*'There’s overuse of antibiotics, and there’s not a clear understanding of whether it’s a viral or bacterial infection, it’s very difficult for a GP to not prescribe when they come across an ill patient like me.'* (PT-02-001-005)

*'A lot of them were so positive* […] *somebody actually said, "I don't want you to give me antibiotics, I'd like to come up for the test my friend had."'* (GP-02-001)

As described above, some nursing staff reported variation among prescribing clinicians with regard to how the test influenced their clinical management. The GPs interviewed all stated that they recognised the importance of using the test as an adjunct to clinical assessment, and some felt the test was most useful as a rule-out tool:

*'Medicine isn't just about a test* […] *medicine is treating the patient holistically* […] *it did sway our point of view* [… but] *we used it as an adjunct to our clinical examination.'* (GP-07-004)

*'I think it’s more reinforcing a decision not to prescribe* […] *I think it’s safe, if used in that way.'* (GP-10-006)

Sometimes where an unexpected (negative) result was obtained, the clinician switched to a delayed prescribing approach, which seemed to be acceptable to patients:

*'I did get prescribed antibiotics but I think the GP had already confirmed via this test that it wasn’t an infection, but she gave me some* [delayed antibiotics] *just in case.'* (PT-05-001-011)

## Discussion

### Summary

Patients’ and HCPs’ views about use of the test were mostly positive. They felt it was a useful tool for guiding antibiotic use and supporting shared decision making. Most felt it would be feasible to integrate into routine consultations, but some reported initial difficulties with blood collection and interpreting results, which impacted on usability. Some patients’ reactions to negative test results suggest the need for better communication about LRTIs and antibiotics alongside use of the test.

### Strengths and limitations

The study had low representation of ethnic minorities (reflective of the area in South England where recruitment took place), as well as parents of children. Two-thirds of the general practices were in areas of high socioeconomic status (Index of Multiple Deprivation [IMD] decile 9 or 10), and 90% of the HCP participants were female. Patients and HCPs with an interest in research and/or with views on use of POCT are more likely to have volunteered to participate, and a number of the patients interviewed had a healthcare-related background, which may have influenced our findings. A single researcher, with a healthcare background but without prior knowledge of FebriDx, performed the qualitative interviews and led the data analysis. Finally, a strength of the study was that recruiting general practices were given flexibility on how to integrate the test into their practices, which increased the variety of experiences and is likely to better reflect ‘real-world’ use of the device.

### Comparison with existing literature

While POCT for infections in primary care have been broadly well-received in studies, the level of uptake into routine UK primary care remains low,^10,12,13,35^ and a lack of feedback and input from clinicians into their design and implementation has been cited as a key barrier.^6,8,9,36^ To our knowledge, only one small retrospective observational study has examined the use of FebriDx in primary care,^14^ and no previous studies have explored the views of patients or clinicians towards FebriDx in any setting. As a standalone, hand-held test, FebriDx may have noteworthy advantages over bulky and expensive POCT devices requiring separate desktop analysers. Recent UK qualitative studies of POCT devices measuring CRP have shown that barriers to adoption include the burdens associated with the maintenance and quality assurance of analysers (potentially needing to involve external laboratories).^9,10^ There are also potential impacts on working practices and clinic flow, particularly given that clinicians in primary care typically work in individual rooms.^9,10^ Furthermore, high up-front costs are a major barrier, particularly in the absence of a national funding and reimbursement policy (such as has been employed in some European countries).^9^

Our findings that clinicians and patients value shared decision making, are consistent with previous studies of using CRP POCT.^10,37–39^ This approach (especially when complemented with visual decision aids) can have a positive impact on both patient satisfaction and health outcomes.^38,40–44^ Our study also highlights the importance of communication, and that effectively counselling patients (both before and after testing) is paramount to ensure patient understanding and satisfaction, as well as facilitating appropriate antibiotic use. Patients’ knowledge and expectations (as well as clinicians’ assumptions about these) have a central role in the prescribing process, and previous studies have demonstrated that ineffective communication contributes to unnecessary antibiotic use.^45–55^ Explaining the difference between bacterial and viral infection, the role of biomarkers (including CRP and MxA), and the possible results and their meaning and implications (including a ‘negative’ result) is crucial; particularly as patients may have poor understanding and expect to receive antibiotics, especially if they have previously.^46,51,54,56,57^ Furthermore, conveying empathy and use of positive language without being dismissive (for example, *'I am pleased to say that the results suggest you won’t benefit from antibiotics'*) may improve patient satisfaction, especially in cases where patients might have been expecting antibiotics.^58–63^

### Implications for research and practice

Together with the results of the overarching feasibility study (which showed that FebriDx use was associated with a substantial reduction in prescribing intentions), our findings support a funding application for a fully powered trial to assess the impact of FebriDx in primary care on antibiotic use. A future trial should also assess impact on symptoms and safety (including re-attendance rates), as well as cost-effectiveness, particularly as costs of implementation are a key barrier to routine adoption of POCT.^9,10^

Some HCPs reported initial difficulties in using the test, suggesting that training and opportunities to gain experience are important. Training on interpreting FebriDx results and effectively communicating these to patients (particularly negative results), as well as wider education on the nature of LRTIs and antibiotic use, is also likely to be important to ensure patient satisfaction and understanding. Helpful online training resources include the Royal College of General Practitioners TARGET antibiotics toolkit, which includes advice and training on discussing antibiotics and LRTIs with patients, as well as patient information leaflets.^64^

Future research should also assess the implementation of FebriDx outside the traditional GP practice setting, such as respiratory infection hubs set up during peak periods,^65^ as well as nursing homes and out-of-hours care (associated with some of the highest rates of inappropriate antibiotic use),^66,67^ and community pharmacies.^68–72^

In conclusion, FebriDx may be a useful tool to guide antibiotic prescribing for LRTI in primary care and combat growing antimicrobial resistance. Training and practice on using the test and effective communication are likely to be critical elements in ensuring patient understanding and satisfaction, and successful adoption.

## Data

The dataset relied on in this article is available from the corresponding author on reasonable request.
